# Supplementary material for: `Friend or foe’ and decision making initiative in complex conflict environments
Source: PLoS One. 2023 Feb 6;18(2):e0281169. doi: 10.1371/journal.pone.0281169 (PMC9901805; doi:10.1371/journal.pone.0281169)
Supplement: S1 File — (PDF) [file pone.0281169.s001.pdf]

Mathematical appendices

Mathew Zuparic[1*], Sergiy Shelyag[2], Maia Angelova[2], Ye Zhu[2], Alexander Kalloniatis[1]

1 Defence Science and Technology Group, Canberra, ACT 2600, Australia
2 School of IT, Deakin University, Melbourne, VIC 3125, Australia

* mathew.zuparic@defence.gov.au

Appendix A: Mathematical properties of the model

We examine model fixed points with the Kuramoto-Sakaguchi component simplified via Equations (23–27) in the main text. Thus the defining equations (reproduced for convenience) are

$$\dot{B} = -\kappa_{RB} \cdot \frac{1-(\sin\Delta_{BG}+\sin\Delta_{GR})}{2} \cdot \frac{g_R}{g_B+\epsilon} \cdot R \cdot \mathcal{H}(B) \tag{37}$$

$$\dot{R} = -\kappa_{BR} \cdot \frac{1+\sin\Delta_{BG}+\sin\Delta_{GR}}{2} \cdot \frac{g_B}{g_R+\epsilon} \cdot B \cdot \mathcal{H}(R) \tag{38}$$

$$\dot{g}_B = -\kappa_{GB} \cdot \sin\Delta_{BG} \cdot B \cdot g_B \cdot (G-g_B-g_R) \cdot \mathcal{H}(R) \tag{39}$$

$$\dot{g}_R = \kappa_{GR} \cdot \sin\Delta_{GR} \cdot R \cdot g_R \cdot (G-g_B-g_R) \cdot \mathcal{H}(B) \tag{40}$$

$$\begin{aligned}\dot{\Delta}_{BG} &= \bar{\omega}^B - \bar{\omega}^G - \psi^B_G \sin(\Delta_{BG}-\phi_{BG}) - \psi^G_B \sin\Delta_{BG} \\ &\quad -\psi^B_R A(B)\sin(\Delta_{BG}+\Delta_{GR}-\phi_{BR}) + \psi^G_R \sin\Delta_{GR}, \end{aligned} \tag{41}$$

$$\begin{aligned}\dot{\Delta}_{GR} &= \bar{\omega}^G - \bar{\omega}^R + \psi^G_B \sin\Delta_{BG} - \psi^R_B A(R)\sin(\Delta_{BG}+\Delta_{GR}+\phi_{RB}) \\ &\quad -\psi^G_R \sin\Delta_{GR} - \psi^R_G \sin(\Delta_{GR}+\phi_{RG}), \end{aligned} \tag{42}$$

Setting the left hand sides of Equations (37–42) equal to zero we obtain three sets of fixed points:

$$\begin{aligned}\{B=0, R, g_B, g_R, \Delta^*_{BG}, \Delta^*_{GR}\} &= \{\vec{P}^*_1, \Delta^*_{BG}, \Delta^*_{GR}\} \\ \{B, R=0, g_B, g_R, \Delta^*_{BG}, \Delta^*_{GR}\} &= \{\vec{P}^*_2, \Delta^*_{BG}, \Delta^*_{GR}\} \\ \{B, R, g_B=0, g_R=0, \Delta^*_{BG}, \Delta^*_{GR}\} &= \{\vec{P}^*_3, \Delta^*_{BG}, \Delta^*_{GR}\}\end{aligned} \tag{43}$$

The first four entries of the lists in Eq.(43) ensure that the Lanchester, Equations (37–38), and Lotka-Volterra, Equations (39–40), components are fixed — generally only requiring one or two of the four variables to be zero. The last two entries ensure that the Kuramoto-Sakaguchi component, Equations (41–42), is either fixed, or in a limit cycle. The fixed points of Equations (41–42), without feedback terms A, were explored in depth in [1], shown to be the roots of a sixth-order polynomial — thus analytic solutions are out of reach. Nevertheless, stability analysis reveals possible system behaviours based on the fixed point values. Specifically, substituting $\Delta_{BG} = \Delta^*_{BG} + \delta_1$ and $\Delta_{GR} = \Delta^*_{GR} + \delta_2$ into Equations (41–42), where Δ^*_{BG} and Δ^*_{GR} are polynomial roots, and the time-dependant perturbations δ_1 and δ_2 are small, *i.e.* $\delta_1^2 \approx \delta_1\delta_2 \approx \delta_2^2 \approx 0$, we obtain

$$\dot{\delta}_1 = \beta_{11}\delta_1 + \beta_{12}\delta_2, \quad \dot{\delta}_2 = \beta_{21}\delta_1 + \beta_{22}\delta_2, \tag{44}$$

where:

$$\begin{aligned}\beta_{11} &= -\psi^B_G \cos(\Delta^*_{BG}-\phi_{BG}) - \psi^G_B \cos\Delta^*_{BG} - \psi^B_R A(B)\cos(\Delta^*_{BG}+\Delta^*_{GR}-\phi_{BR}) \\ \beta_{12} &= \psi^G_R \cos\Delta^*_{GR} - \psi^B_R A(B)\cos(\Delta^*_{BG}+\Delta^*_{GR}-\phi_{BR}) \\ \beta_{21} &= \psi^G_B \cos\Delta^*_{BG} - \psi^R_B A(R)\cos(\Delta^*_{BG}+\Delta^*_{GR}+\phi_{RB}) \\ \beta_{22} &= -\psi^G_R \cos\Delta^*_{GR} - \psi^R_G \cos(\Delta^*_{GR}+\phi_{RG}) - \psi^R_B A(R)\cos(\Delta^*_{BG}+\Delta^*_{GR}+\phi_{RB})\end{aligned} \tag{45}$$

and the dynamics of force values B and R are assumed to be much slower than the perturbations δ_1 and δ_2. Thus, the eigenvalues of the linearised Kuramoto-Sakaguchi system which classify dynamic behaviour are:

$$\lambda_\pm = \frac{\beta_{11}+\beta_{22}}{2} \pm \sqrt{\frac{(\beta_{11}+\beta_{22})^2}{4} + \beta_{12}\beta_{21}}. \tag{46}$$

Asymptotically stable fixed points (stable nodes) for Equations (41–42) exist when the real components of both eigenvalues $\lambda_\pm$ are negative, and the imaginary components are zero. In the considered region of μ_R and μ_B with fixed feedback values, $A(B) = A(R) = 1$, we numerically observe asymptotically stable fixed point, and unstable behaviour of the system — see Figure 10. However, in the $[-1, 1)$ region, asymptotically stable limit cycle solutions also exist. The appearance of force size values B and R in the eigenvalues means that long-time behaviour can qualitatively change as the force sizes vary. This behaviour, and its implications on model outcomes, is discussed in Figure 3 in the main body of this work.

To examine the fixed points of the Lanchester and Volterra-Lotka components, let $\vec{P} = \{B, R, g_B, g_R\}$, then Equations (37–40) can be re-expressed as

$$\dot{\vec{P}} = \vec{u}\left(\vec{P}\right) = \left\{u_B\left(\vec{P}\right), u_R\left(\vec{P}\right), u_{g_B}\left(\vec{P}\right), u_{g_R}\left(\vec{P}\right)\right\}, \tag{47}$$

where the Kuramoto-Sakaguchi components are assumed to be autonomous inputs due to the time-scale differences. Thus, expanding Equation (47) near the fixed points, $\vec{P} \approx \vec{P}^* + \vec{\delta}$, where the four $\vec{P}^*$ values are given explicitly in Equation (43), and keeping only linear terms in $\vec{\delta}$, we obtain,

$$\dot{\vec{\delta}} = \begin{pmatrix} \frac{\partial}{\partial B}u_B & \frac{\partial}{\partial R}u_B & \frac{\partial}{\partial g_B}u_B & \frac{\partial}{\partial g_R}u_B \\ \frac{\partial}{\partial B}u_R & \frac{\partial}{\partial R}u_R & \frac{\partial}{\partial g_B}u_R & \frac{\partial}{\partial g_R}u_R \\ \frac{\partial}{\partial B}u_{g_B} & \frac{\partial}{\partial R}u_{g_B} & \frac{\partial}{\partial g_B}u_{g_B} & \frac{\partial}{\partial g_R}u_{g_B} \\ \frac{\partial}{\partial B}u_{g_R} & \frac{\partial}{\partial R}u_{g_R} & \frac{\partial}{\partial g_B}u_{g_R} & \frac{\partial}{\partial g_R}u_{g_R} \end{pmatrix}_{\vec{P}\to\vec{P}^*} .\vec{\delta}, \tag{48}$$

which is a 4-dimensional generalisation of Equation (44). Applying the values of $\vec{P}^*$ from Equation (43), the corresponding eigenvalues are

$$\begin{aligned} \{\lambda_1,\lambda_2,\lambda_3,\lambda_4\}_{\vec{P}\to\vec{P}_1^*} &= \left\{-\alpha_{RB}\frac{g_R}{g_B+\epsilon}R\lim_{B\to0^+}\mathcal{H}'(B),0,0,0\right\} \\ \{\lambda_1,\lambda_2,\lambda_3,\lambda_4\}_{\vec{P}\to\vec{P}_2^*} &= \left\{0,-\alpha_{BR}\frac{g_B}{g_R+\epsilon}B\lim_{R\to0^+}\mathcal{H}'(R),0,0\right\} \\ \{\lambda_1,\lambda_2,\lambda_3,\lambda_4\}_{\vec{P}\to\vec{P}_3^*} &= \{0,0,\alpha_{GB}B\cdot G\cdot\mathcal{H}(R),\alpha_{GR}R\cdot G\cdot\mathcal{H}(B)\}. \end{aligned} \tag{49}$$

Thus, the fixed points $\vec{P}_1^*$ and $\vec{P}_2^*$ are marginally stable, having only one negative eigenvalue, and the remaining three being zero. Additionally, if either $\alpha_{GB} > 0$ and/or $\alpha_{GR} > 0$, then $\vec{P}_3^*$ is asymptotically unstable.

The defining Lanchester equations possess only monotonic trajectories, due to the right hand sides of Equations (37–38) never being positive. Therefore, chaotic Lanchester trajectories for combat force outcomes are not possible in this model [2]. Addressing equivalent properties the Kuramoto-Sakaguchi component, Equations (41–42) are a generalisation of the 3-node Kuramoto model, whose complicated bifurcation properties were explored in Section 4 of [3]. Figure 10 of this work presents numerical bifurcation analysis of Equations (41–42) with simplified feedback terms $A(B) = A(R) = 1$. The left panel shows one-dimensional bifurcation analysis results of Δ_{BG} and Δ_{BR} as μ_B varies. Fixed point solutions, corresponding to eigenvalues in Equation (46) satisfying

$$Re\{\lambda_\pm\} < 0 \text{ and } Im\{\lambda_\pm\} = 0, \tag{50}$$

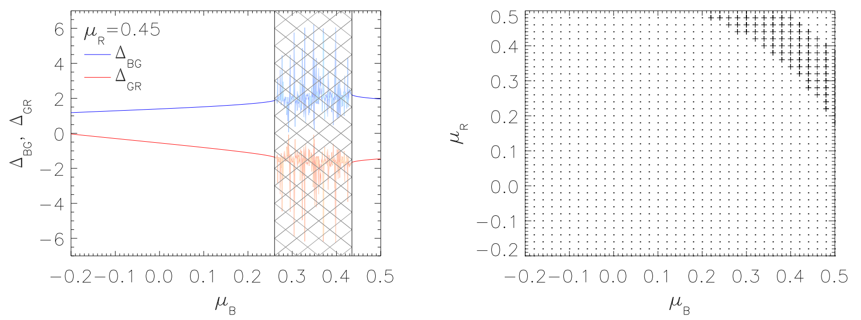

Fig 10. Numerical bifurcation analysis of the Kuramoto-Sakaguchi component of the model, given in Equations (41–42) with feedback terms $A(B) = A(R) = 1$. Left plot shows a one-dimensional bifurcation diagram for Δ_{BG} (blue) and Δ_{GR} (red). The solution regions, corresponding to fixed points, are shown as solid curves. The unstable region is shown as a hatched area. Right plot shows a two-dimensional bifurcation diagram for the range of μ_B and μ_R as in the main body of the paper. Crosses and dots correspond to the unstable and fixed point behaviours of the system, respectively. The unstable region corresponds to the steady state condition of Equation (50) not being adhered to (see the left plot of Figure 9 in the main body).

are given by solid lines. Equivalently, unstable regions, which do not abide by Equation (50), are denoted by the hatched area. The right plot shows a two-dimensional bifurcation diagram of the system as a function of μ_B and μ_R. Dots denote fixed point solutions of Δ_{BG} and Δ_{GR} which obey the stability condition of Equation (50). Correspondingly, crosses denote unstable behaviour regions of Δ_{BG} and Δ_{GR}, corresponding to parameter values where steady state assumptions of Equation (50) are not valid. Additionally, this behaviour was displayed in Figure 9 of the main body of this work.

Appendix B: Derivation of Equation 32

This Appendix provides the steps to reproduce Equation 32 in the main body. Summing Equations (41–42), we obtain the following expression for the dynamics of $\Delta_{BR} \equiv \Delta_{BG} + \Delta_{GR}$:

$$\dot{\Delta}_{BR} = \bar{\omega}^B - \bar{\omega}^R - \psi_R^B \left[A(B)\sin(\Delta_{BR} - \phi_{BR}) + A(R)\sin(\Delta_{BR} + \phi_{RB})\right] \\ -\psi_G^B \sin(\Delta_{BG} - \phi_{BG}) + \psi_R^G \sin(\Delta_{GR} + \phi_{RG}) \quad (51)$$

The terms Δ_{BG} and Δ_{GR} in Equation (51) cannot be combined to create a function solely of Δ_{BR}. In order to proceed, we approximate the terms Δ_{BG} and Δ_{GR} in Equation (51) by equating them to the valid roots — labeled Δ^*_{BG} and Δ^*_{GR} — of the system

$$0 = \bar{\omega}^B - \bar{\omega}^G - \psi_G^B \sin(\Delta_{BG} - \phi_{BG}) - \psi_B^G \sin\Delta_{BG} - \\ -\psi_R^B \sin(\Delta_{BG} + \Delta_{GR} - \phi_{BR}) + \psi_R^G \sin\Delta_{GR} \quad (52)$$

$$0 = \bar{\omega}^G - \bar{\omega}^R + \psi_B^G \sin\Delta_{BG} - \psi_B^R \sin(\Delta_{BG} + \Delta_{GR} + \phi_{RB}) - \\ -\psi_R^G \sin\Delta_{GR} - \psi_G^R \sin(\Delta_{GR} + \phi_{RG}) \quad (53)$$

The combined roots of Equations (52–53) are the fixed points of Equations (41–42) with the attenuation term A set to unity. The valid roots of Equations (52–53) were shown to have no imaginary-components, in addition to negative real-components of the

corresponding Lyapunov exponents [1]. Roots satisfying these conditions were shown to be the steady-state solutions to Equations (41–42) with no attenuation term, *i.e.*

$$\Delta_{BG}(t)|_{\substack{t\to\infty \\ A=1}} \equiv \Delta^*_{BG}, \quad \Delta_{GR}(t)|_{\substack{t\to\infty \\ A=1}} \equiv \Delta^*_{GR} \tag{54}$$

Thus, Equation (51) for $\dot{\Delta}_{BR}$ approximates to

$$\dot{\Delta}_{BR} \approx \chi + S(B,R)\cos\Delta_{BR} - C(B,R)\sin\Delta_{BR} \tag{55}$$

Assuming that the dynamics of B and R are slow compared to Δ_{BR} (i.e. decision-related time-scales are faster than combat outcomes), we can take B, R as time-independent and integrate Equation (51) to give an approximate time-dependent solution [4]

$$\Delta_{BR}(t) \approx 2\tan^{-1}\left[\frac{C(B,R) - \sqrt{K(B,R)}\tanh\left(\frac{t+const}{2}\sqrt{K(B,R)}\right)}{\chi - S(B,R)}\right] \tag{56}$$

$$\text{for} \quad K(B,R) = S^2(B,R) + C^2(B,R) - \chi^2, \tag{57}$$

and *const* in Equation (56) encodes the initial condition. In the absence of feedback with B and R, Δ_{BR} only displays 2 types of behaviour: obtaining a steady state value after a brief transient, or bounded periodic limit cycles. As the dynamics of B and R are slow compared to Δ_{BR}, the dynamics of Δ_{BR} is assumed to follow a similar trajectory to what was seen in [1], with slight modifications due to dynamic B and R, captured in the form of C and S in Equation (33) in the main text. Thus we focus on the steady-state form of Δ_{BR}, obtained by the $t \to \infty$ limit in Equation (56)

$$\Delta_{BR}(B,R) \equiv \Delta_{BR}(t \to \infty), \tag{58}$$

which is the form given in Equation (32) in the main body.

Appendix C: Line integral formulation and approximation

Approximating each B and R trajectory in Equation (32) of the main text via linear paths requires using the final value of the engagement (given in the top-left panel in Figure 4 of the main text), labelled as $|B(T_{end}) - R(T_{end})| \equiv Z$. Assuming B and R decay linearly, they are parameterised by

$$R = \frac{R_0(B-Z)}{B_0 - Z} \quad \text{for Blue victory,} \quad B = \frac{B_0(R-Z)}{R_0 - Z} \quad \text{for Red victory.} \tag{59}$$

Assuming a linear relation between B and R via Equation (59) simplifies the line integral to

$$\int_{\mathcal{C}} ds = \begin{cases} \sqrt{1+\left(\frac{R_0}{B_0-Z}\right)^2}\int_{B_0}^{Z} dB & \text{for Blue victory} \\ \sqrt{1+\left(\frac{B_0}{R_0-Z}\right)^2}\int_{R_0}^{Z} dR & \text{for Red victory} \end{cases}. \tag{60}$$

Thus, substituting the analytic form for Δ_{BR} given by Equation (32) of the main text into Equation (36) of the main text, with linearised B and R trajectories yields

$$\left\langle \tan\frac{\Delta_{BR}}{2}\right\rangle \approx \begin{cases} \int_{B_0}^{Z} \frac{dB}{Z-B_0}\left(\frac{C\left(B,\frac{R_0(B-Z)}{B_0-Z}\right) - \sqrt{K\left(B,\frac{R_0(B-Z)}{B_0-Z}\right)}}{\chi - S\left(B,\frac{R_0(B-Z)}{B_0-Z}\right)}\right) & \text{for Blue victory} \\ \int_{R_0}^{Z} \frac{dR}{Z-R_0}\left(\frac{C\left(\frac{B_0(R-Z)}{R_0-Z},R\right) - \sqrt{K\left(\frac{B_0(R-Z)}{R_0-Z},R\right)}}{\chi - S\left(\frac{B_0(R-Z)}{R_0-Z},R\right)}\right) & \text{for Red victory} \end{cases} \tag{61}$$

where K is defined in Equation (57). To obtain the value of $\langle \sin \Delta_{BR} \rangle$ from Equation (61) we apply the approximation

$$\langle \sin \Delta_{BR} \rangle \approx \sin \left(2 \tan^{-1} \left\langle \tan \frac{\Delta_{BR}}{2} \right\rangle \right). \tag{62}$$

Equation (62) is the approximate form applied to generate the left panel of Figure 9 in the main text. Additionally, it is possible to obtain explicit solutions of the Blue and Red victory integrals given by Equation (61). Focusing on either Blue or Red victory, the integral in question becomes

$$\underbrace{\int_{F_0}^{Z} \frac{dF}{Z - F_0} \left(\frac{C_X^F F - C_Y^F}{\chi - S_X^F F - S_Y^F} \right)}_{INT_1^F} - \underbrace{\int_{F_0}^{Z} \frac{dF}{Z - F_0} \left(\frac{\sqrt{(C_X^F F - C_Y^F)^2 + (S_X^F F + S_Y^F)^2 - \chi^2}}{\chi - S_X^F B - S_Y^F} \right)}_{INT_2^F} \tag{63}$$

where $F = \{B, R\}$ signifies the victor of the engagement, and

$$\begin{aligned} C_X^B &= \psi_R^B \left(\frac{\cos \phi_{BR}}{B_0} + \frac{\cos \phi_{RB}}{B_0 - Z} \right) \quad & C_Y^B &= \psi_R^B \frac{Z \cos \phi_{RB}}{B_0 - Z} \\ S_X^B &= \psi_R^B \left(\frac{\sin \phi_{BR}}{B_0} - \frac{\sin \phi_{RB}}{B_0 - Z} \right) \quad & S_Y^B &= \psi_R^B \frac{Z \sin \phi_{RB}}{B_0 - Z}, \end{aligned} \tag{64}$$

$$\begin{aligned} C_X^R &= \psi_R^B \left(\frac{\cos \phi_{RB}}{R_0} + \frac{\cos \phi_{BR}}{R_0 - Z} \right) \quad & C_Y^R &= \psi_R^B \frac{Z \cos \phi_{BR}}{R_0 - Z} \\ S_X^R &= \psi_R^B \left(\frac{\sin \phi_{RB}}{R_0} - \frac{\sin \phi_{BR}}{R_0 - Z} \right) \quad & S_Y^R &= -\psi_R^B \frac{Z \sin \phi_{BR}}{R_0 - Z}. \end{aligned} \tag{65}$$

Each of the integrals in Equation (63) can be computed explicitly, revealing

$$INT_1^F = \frac{[C_Y^F S_X^F + C_X^F (S_Y^F - \chi)] \ln \left(\frac{\chi - S_X^F Z - S_Y^F}{\chi - S_X^F F_0 - S_Y^F} \right) - C_X^F S_X^F (Z - F_0)}{(Z - F_0)(S_X^F)^2} \tag{66}$$

$$\begin{aligned} INT_2^F = \Bigg[& \frac{S_X^F \sqrt{(C_X^F F - C_Y^F)^2 + (S_X^F F + S_Y^F)^2 - \chi^2}}{(Z - F_0)(S_X^F)^2} + [C_X^F C_Y^F S_X^F - \chi (S_X^F)^2 \\ & + (C_X^F)^2 (S_Y^F - \chi)] \frac{\tanh^{-1} \left\{ \frac{(C_X^F)^2 F - C_X^F C_Y^F + S_X^F (S_X^F F + S_Y^F)}{\sqrt{(C_X^F)^2 + (C_Y^F)^2} \sqrt{(C_X^F F - C_Y^F)^2 + (S_X^F B + S_Y^F)^2 - \chi^2}} \right\}}{(Z - F_0)(S_X^F)^2 \sqrt{(C_X^F)^2 + (C_Y^F)^2}} + [C_X^F (\chi - S_Y^F) \\ & - C_Y^F S_X^F] \frac{\tanh^{-1} \left\{ \frac{C_X^F C_Y^F (\chi + S_X^F F - S_Y^F) + F (C_X^F)^2 (S_Y^F - \chi) - S_X^F [(C_Y^F)^2 + \chi (S_X^F F + S_Y^F - \chi)]}{[C_Y^F S_X^F + C_X^F (S_Y^F - \chi)] \sqrt{(C_X^F F - C_Y^F)^2 + (S_X^F F + S_Y^F)^2 - \chi^2}} \right\}}{(Z - F_0)(S_X^F)^2} \Bigg]_{F \to F_0}^{F \to Z}. \end{aligned} \tag{67}$$

References

1. Zuparic M, Angelova M, Zhu Y and Kalloniatis A. Adversarial decision strategies in multiple network phased oscillators: The Blue-Green-Red Kuramoto-Sakaguchi model. *Commun. Nonlinear Sci. Numer. Simul.* 2021;**95**:105642.

2. Hirsch M. On the nonchaotic nature of monotone dynamical systems. *Eur. J. Pure Appl. Math* 2019;**12**(3):680–8.

3. Maistrenko Y., Popovych O. and Tass P. Chaotic attractor in the Kuramoto model. *Int J Bifurcat Chaos* 2005;**15**(11):3457—66.

4. Kalloniatis A.C. and Zuparic M. Fixed points and stability in the two-network frustrated Kuramoto model. *Physica A* 2016;**447**:21–35.
